# Challenge infection model for MERS-CoV based on naturally infected camels

**DOI:** 10.1186/s12985-020-01347-5

**Published:** 2020-06-17

**Authors:** Naif Khalaf Alharbi, Osman H. Ibrahim, Ali Alhafufi, Samy Kasem, Ali Aldowerij, Raed Albrahim, Ali Abu-obaidah, Ali Alkarar, Faisal Altaib Bayoumi, Ali Mohammed Almansour, Musaad Aldubaib, Hail M. Al-Abdely, Hanan H. Balkhy, Ibrahim Qasim

**Affiliations:** 1 Department of Infectious Disease Research, King Abdullah International Medical Research Center, Riyadh, Saudi Arabia; 2 King Saud bin Abdulaziz University for Health Sciences, Riyadh, Saudi Arabia; 3 Ministry of Environment, Water and Agriculture (MEWA), Riyadh, Saudi Arabia; 4 Department of Virology, Faculty of Veterinary Medicine, Kafrelsheikh University, El Geish Street, Kafrelsheikh, 33516 Egypt; 5 College of Agriculture and Veterinary Medicine, Qassim University, Qassim, Saudi Arabia; 6 Ministry of Health, Riyadh, Saudi Arabia; 7 Internal Medicine Department, King Faisal Specialist Hospital and Research Center, Riyadh, Saudi Arabia; 8 Department of Infection Prevention and Control, Ministry of National Guard - Health Affairs, Riyadh, Saudi Arabia

**Keywords:** MERS-CoV, Dromedary camels, Seroprevalence, Saudi Arabia, Vaccine efficacy, Challenge model

## Abstract

**Background:**

Middle East Respiratory Syndrome coronavirus (MERS-CoV) is an emerging virus that infects humans and camels with no approved antiviral therapy or vaccine. Some vaccines are in development for camels as a one-health intervention where vaccinating camels is proposed to reduce human viral exposure. This intervention will require an understanding of the prior exposure of camels to the virus and appropriate vaccine efficacy studies in camels.

**Methods:**

We conducted a cross sectional seroprevalence study in young dromedary camels to determine the rate of MERS-CoV seropositivity in young camels. Next, we utilised naturally infected camels as a natural challenge model that can be used by co-housing these camels with healthy naive camels in a ratio of 1 to 2. This model is aimed to support studies on natural virus transmission as well as evaluating drug and vaccine efficacy.

**Results:**

We found that 90% of the screened camels have pre-existing antibodies for MERS-CoV. In addition, the challenge model resulted in MERS-CoV transmission within 48 h with infections that continued for 14 days post challenge.

**Conclusions:**

Our finding suggests that the majority of young dromedary camels in Saudi Arabia are seropositive and that naturally infected camels can serve as a challenge model to assess transmission, therapeutics, and vaccine efficacy.

## Background

Middle East Respiratory Syndrome Coronavirus (MERS-CoV) was identified in 2012 from a pneumonic patient who subsequently died, in the Kingdom of Saudi Arabia (KSA) [1]. Since its emergence, the virus has infected more than 2450 individuals in 27 countries [2]. Outbreaks occurred mainly in the Arabian Peninsula in large crowded hospitals with one large outbreak in the Republic of Korea [3]. To date, bats were suggested, but have not been confirmed, as the virus natural reservoir with some suggestive experimental data [4], whilst dromedary camels are the only confirmed intermediate animal host. 54.9% of human primary cases have reported contact with camels [5] and the index patient in the Korean outbreak traveled back from the Gulf countries where MERS-CoV is endemic and circulating in dromedary camels [6]. Dromedaries in Africa and Arabia have a high rate of seroprevalence ranging from 74 to 100% [7–11]. More importantly, these animals seem to have been infected with MERS-CoV by as early as 1983 according to serological data on archived dromedary sera. Samples from several countries in Africa and Arabia, collected in different years between 1983 and 2010, were seropositive for MERS-CoV with a range of seroprevalence between 29 and 97% [8, 12–16]. Moreover, a recent study looking at dromedaries associated with confirmed human cases in Saudi Arabia found that 70% of these camels were seropositive and that viral RNA could be detected in 12% of these camels [6, 17].

Adult camels and older calves are more likely to be seropositive as compared to younger calves; also viral RNA is more likely to be detected in younger, seronegative, calves [6, 10, 17, 18]; however, this tendency was not significantly different in other studies [9]. Calves initially possess maternal anti-MERS-CoV antibodies that wane by five to six months of age [19] leaving them susceptible to infection. However, MERS-CoV reinfection into seropositive camels has been reported [20], indicating that pre-existing immunity does not prevent new MERS viral infection in camels although the viral load may be reduced. Currently, there is no approved antiviral therapy or vaccine against MERS-CoV in humans or camels. Therefore, vaccines against camel MERS-CoV infections are being developed with the aim of reducing viral transmission and introduction into humans [21]. Three vaccine candidates have been evaluated in dromedaries so far; a DNA based vaccine [22], a poxviral vectored vaccines [23], and an adenoviral vectored vaccine (data is expected to be available soon from our team). The two published vaccines elicited antibody immune responses in camels and one was partially protective in reducing the viral load in camels upon experimental challenge [23]; however, the experimental challenge might not represent the natural infection in camels. To guide MERS vaccine development in camels, many questions still need to be addressed in experimental settings such as what is the best target population for vaccination (younger calves versus older calves or adult camels)? What is the infectious viral dose that needs to be assessed in vaccine efficacy studies? Here, in an attempt to investigate these questions, we first explore the camel population and density in KSA as well as conducting a cross sectional seroprevalence study in young dromedary to explore the younger camel population as a target for vaccine development. Second, we aimed to utilise MERS-CoV naturally infected camels as a model of challenge for vaccine efficacy studies. Unlike lab experimental challenge, the natural challenge model would mimic the natural setting of MERS-CoV infection in camels. Therefore, this paper reports a cross sectional seroprevalence study in young camel and examines the utility of natural infection in camels as a potential challenge model for MERS vaccine assessment. It also includes information on the dynamics of MERS-CoV natural infection, incubation period, and shedding time.

## Methods

### Pens, camels, personnel, and infection control

A camel research farm was set up 100 km from Riyadh city, remote from urban areas, with double fences and a secured gate. Inside this farm, metal pens were set up with 30 m^2^ in size. Each pen is 150 cm height and has an infection control entry and exit points 10 m away from each other and from the pen. Two surveillance cameras were installed for each pen. Food and water troughs were placed inside each pen, where they can be filled from outside without entering the pens, Figure S1 shows the farm layout.

For the seroprevalence study, serum samples were collected from 362 camels under the age of 2 years in Qassim and Jouf provinces between February and May 2017. Second, five naïve calves (under the age of 2 years) were purchased from different farms in Jouf and transported for more than a 1000 km, using disinfected lorries, to the research farm. These calves were kept in one research pen for an acclimatization period of 3 weeks and were healthy before the experiment was conducted; they were also re-tested for MERS-CoV viral RNA and antibodies and confirmed negative. In addition, three infected camels that were positive by RT-PCR with a Ct value below 25 were purchased from local markets (Riyadh) and mixed with the five naïve calves in one pen to serve as a natural infection model of challenge.

Pen keepers, veterinarians, and drivers were trained for infection control practices by taking a course at the Saudi National Guard hospital, Riyadh, including a fit-test for N95 masks. Each worker used their own specific N95 mask, overall white gown, goggles, head cover, shoe cover, all of which disposable and used once only. The team adhered to utilising the entry and exit point of each pen to ensure infection control; each pen, donning, and doffing area has biohazard waste containers, sharp biohazard containers, 70% Ethanol spray and virucidal ANIOSpray (Laboratoires Anios, France) used by the workers. All staff involved in the study were also screened and confirmed MERS-CoV negative by ELISA and PCR prior to, during, and at the end of the study. Biowaste was collected daily and sent for incineration by the Saudi Gulf Environmental Protection Company (SEPCO). Clean sand from a nearby dunes area was used as pen floors, and new sand were added every 3 months. Pesticide was sprayed over the pen floors before starting the study or when new sand is added.

### Serological assays

The semi-quantitative anti-MERS-CoV ELISA Kits, specific for camels (EUROIMMUN, Lübeck, Germany), were used to detect specific IgG antibodies against MERS CoV in camel sera. The procedures were applied according to the manufacturer’s instructions [6, 13]. Readouts were reported as the ratio of sample optometric density (OD) over the OD of an internal commercial calibrator of the kit. Ratios of ≥1.1 and 0.8–1.1 were assigned as positive and borderline (equivocal), respectively, as recommended by EUROIMMUN. Positive and negative commercial controls provided with the kit were included in each ELISA run.

### RT-qPCR for indirect viral RNA load

Nasal swabs from camels were collected in virus transport media (VTM, from UTM, COPAN). The viral RNA was extracted using MagNA Pure 96 DNA and Viral NA Small Volume Kit and MagnaPure96 machine (Roche Diagnostics, USA). Extracted RNA samples were used to set up one step RT-qPCR using Modular dx Corona MERS-CoV UpE gene and ORF1a gene kit [24–26]; and the PCR was then run using LightCycler480II (Roche Diagnostics, USA). Samples were considered positive if both UpE and ORF1a amplicons were detected. Ct value of 37 was considered the positive cut-off; and negative or undetectable RNA were arbitrarily given a Ct value of 40 to be presented in a graph.

### Statistical analysis

All data were plotted using Graphpad Prism software. No statistical testing was performed; a simple calculation of percentage for the seropositive camels was applied.

## Results

### Camel population and antibody immune responses in young dromedary in Saudi Arabia

**Fig. 1:**
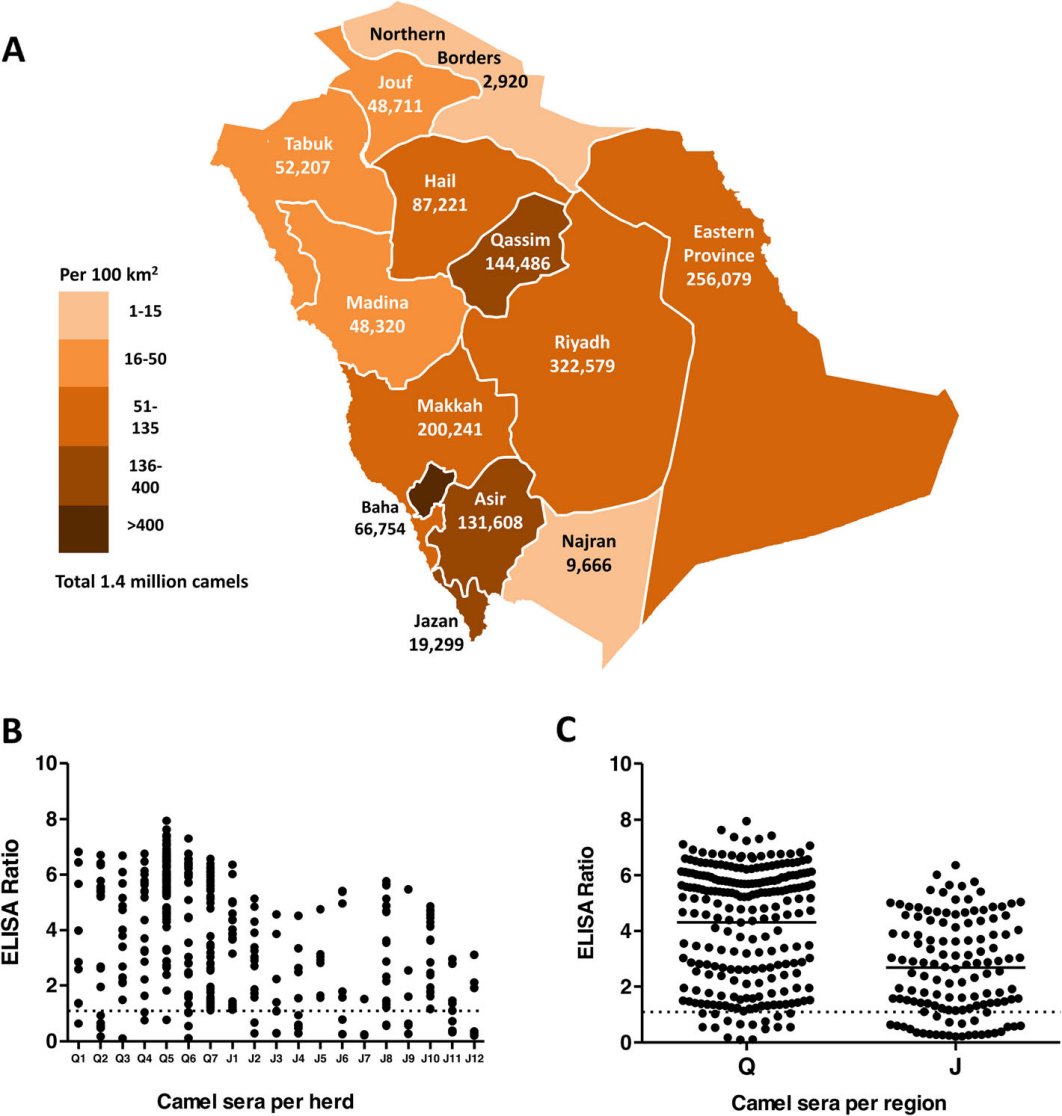
Screening of young camels for MERS-CoV antibodies. **a:** A representative map of KSA showing the number of camels per province, written underneath province names. Density of colour represents the camel number per 100 km^2^ in each province. **b** and **c**: 362 dromedary calves below the age of 2 years were screened for anti-S1 antibodies using EUROIMMUN ELISA kits. ELISA Ratio of > 1.1 is positive, < 0.8 is negative, between 0.8 and 1.1 is equivocal. Dotted line represents the assay positive cutoff of 1.1. Seven different herds and farms were screened in Qassim (Q) province and twelve in Jouf (J) province

We utilised our records at the Saudi Ministry of Environment, Water and Agriculture (MEWA) to present the latest available census on dromedary camels in KSA, which was also available by the Saudi General Authority for statistics [27]. In 2016, there were 1.4 million camels in KSA, distributed across the country but mainly in the Eastern, Riyadh, Makkah, and Qassim provinces, Fig. 1a.

Based on previous studies, camels in KSA are largely seropositive for MERS-CoV, especially older camels. Therefore, to assess immune responses in young dromedaries as a potential target for vaccine development, calves and camels below the age of two years old were screened. 362 camels from nineteen different herds (or farms) in the province of Qassim (Q), *n* = 233, and Jouf (J), *n* = 129, were tested using anti-MERS spike subunit 1 based ELISA kits between February and May 2017. Only 36 samples (12 in Q and 24 in J) did not show antibodies above the equivocal cutoff, a ratio of 0.8, therefore these animals were considered seronegative (naive). This finding indicates that MERS-CoV seroprevalence in young camels, in these provinces at the time, was 90%. In particular, the seroprevalence rate in young camels was 94.5 and 77% in Qassim and Jouf respectively. The seronegative camels were identified in most of the screened herds (or farms), and were not clustered in a specific herd (Table S1). The remaining samples were positive, including six samples that were in the equivocal range, from 0.8 to 1.1 ratios, Fig. 1b, c and Table S1. The antibody ratios of positive samples span from 1.1 to 7.95. The high ratios could indicate recent infections or a boost re-infection in some camels.

### Assessment of MERS-CoV natural infection in dromedaries as a challenge model

**Fig. 2:**
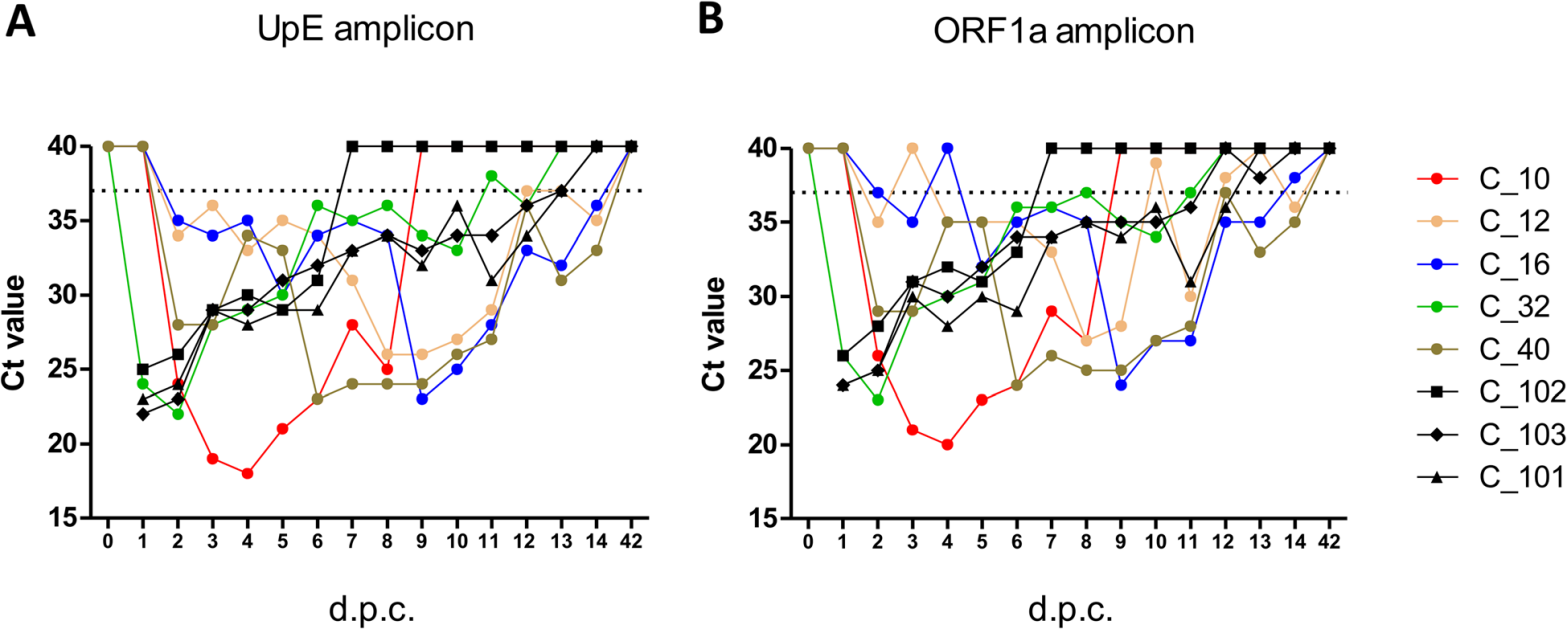
MERS-CoV natural infection in dromedaries as a challenge model Five calves (closed circle symbols) were mixed with three naturally infected camels (black symbols). Nasal swabs were assessed for the presence of MERS-CoV UpE (**a**) and ORF1a (**v**) amplicons by RT-qPCR for 14 (and at 42) d.p.c. Pre-challenge Ct values for the naïve calves were also included (0 d.p.c.). Ct values are shown for individual camels. Dotted line represents the cutoff Ct value of 37. A value of 40 were given to all undetectable (negative) samples

To assess the utility of MERS-CoV infected camels as a natural challenge model for MERS vaccine efficacy studies, three camels (not age-selected) were confirmed to have MERS-CoV infections by RT-qPCR, with Ct values ≤25. Within two days of their lab testing, these camels were transferred and co-housed with five seronegative calves (under 2 years old) in a single pen. Nasal swabs were collected daily for two weeks and then at 42 days post natural challenge (d.p.c.). Two RT-qPCR, specific to the UpE and ORF1a regions of the MERS-CoV genome, showed that all five calves became positive for MERS-CoV infection within 24 to 48 h by both amplicons, Fig. 2 and Table S2. The Ct values of the calves varied at 2 d.p.c. with a difference of around 10 cycle between some calves. This difference in Ct values could indicate a number of possibilities such as that some calves might be more susceptible to MERS-CoV infection, various levels of the MERS-CoV infectious dose in the exposed camels, or a camel behavior that could increase the likelihood of virus transmission. The Ct values in samples from camels number 12 and 16 were in the positive range (Ct ≤ 35 for UpE gene) at 2 to 6 d.p.c., but the Ct values dropped sharply to Ct ≤ ≤ 25 at 9 d.p.c, which could indicate MERS-CoV may require longer time to replicate and establish an infection in some camels or that the continuous exposure of these camels to the virus enhanced the infection, presumably by getting viruses transmitted from different source-camels at different days. The three naturally infected camels seemed to continue to shed virus for most of the experiment period; but with increasing Ct values (decreasing viral load) as the study progressed. One of the naturally infected camels seemed to clear the virus at 7 d.p.c. whereas the virus clearance in the other two was delayed until 12 d.p.c. This indicates that MERS-CoV infection course in camels is approximately two weeks. Of note, all infected camels showed nasal discharge that were concurrent with the viral RNA detection. A follow up testing on all calves and camels showed no sign of infection and undetectable viral RNA at 42 d.p.c., Fig. 2. Overall, these findings indicate that introducing infected camels in a healthy herd, at a ratio of 1:2 could transmit MERS-CoV infection within two days and the infection course would last for at least 14 days.

## Discussion

Dromedary camels are the only confirmed animal host for MERS-CoV and the source of human infections. Here, we report the latest available census on the dromedary population in KSA. We speculate, based on our local knowledge and experience, that the reasons behind camel high population in Eastern, Riyadh, Makkah, and Qassim provinces is the following: Eastern province is largely an empty desert where camel keepers would have more space and natural resources to move their camels; Makkah province would be a large spot and market for animal sacrifice during the sacred Hajj pilgrimage; Riyadh is the most populated province, including the capital city of Riyadh, that has camel markets, camel festivals, and many camel abattoirs and butcher shops; Qassim province is geographically central and it has the biggest camel market in KSA and most probably worldwide.

In this study, we also confirm that seroprevalence rate of MERS-CoV in young dromedaries in two regions of KSA, in 2017, is 90% (Qassim and Jouf had a rate of 94.5 and 77%, respectively), which is in agreement with several previous reports on dromedaries in general (young and adult) in Africa and Arabia [7–11]. Qassim region was selected because it has a large camel market, which is surrounded by camel farms whereas Jouf was selected based on previous data that this region had lower MERS seroprevalence rate [6, 17]. These data have important implications on MERS vaccine development for camels. Although a significant population of camels has pre-existing antibodies to MERS-CoV, these antibodies do not seem to be protective and re-infections in camels have now been documented [20, 28]. Therefore, MERS-CoV vaccines in camels might be designed to boost the natural immune responses in dromedary camels that are usually seropositive. Additionally, naive camels (which are considered a minor population of camels) might require robust vaccination platforms or regimens in order to achieve high and protective titres of antibodies. However, cell-mediated immunity should also be evaluated in naturally infected camels to elucidate reasons behind the re-infection of seropositive camels as well as to guide vaccine development. It is also important to note that with a serpositivity rate of 90%, naïve camels are a small population of camels in Saudi Arabia and it is extremely difficult to source these camels for vaccine studies. Therefore, vaccine studies could be first aimed at seropositive animals to prove that MERS vaccines can, indeed, be efficacious in the camels with pre-existing immunity.

This study is also an attempt to support and enable assessment for MERS-CoV transmission as well as drug and vaccine efficacy in camels where there is no high containment lab for large animals in endemic countries (including KSA). It reports the first natural infection challenge model of MERS-CoV in dromedary camels. RT-qPCR for UpE and ORF1a amplicons, which are the accepted method for documenting MERS infections in humans and camels, was used to confirm infections in camels. We found that infected camels with a Ct value ≤25 co-housed as one-third of a herd in a 30 m^2^ can achieve 100% infection in healthy naive camels within 2 days (as measured by the TR-qPCR). Although we utilised camels with low Ct values, we did not quantify the virus or its RNA levels in these camels; and whether higher Ct values (lower viral load) would achieve the same outcome needs to be confirmed. In addition, although we documented the Ct values of viral RNA for 14 days, it is likely that all camels would have cleared the virus within 3 to 5 weeks, MERS-CoV infectious virus was isolated only within one week post experimental infection in dromedaries whereas the viral RNA was detected for 35 days [29]. Thus, the natural course of MERS infection in camels could be around 2 weeks. The natural infection challenge model of MERS-CoV in camels can be utilised for drug and vaccine efficacy studies, in countries where there is a lack of large animal biosafety level 3 labs. We applied strict infection control measures on the study personnel who were all negative for MERS-CoV RNA and antibodies before, during, and at the end of the study.

Alpacas have been suggested as a surrogate model for MERS vaccine evaluation as they facilitate animal-to-animal transmission and were protected from re-infection [30]; however, when the same vaccine was tested in both alpacas and dromedaries there were differences in immunogenicity and efficacy [31]. All alpacas seroconverted and were completely protected whereas only some dromedaries seroconvert and showed partial protection. Dromedaries that were challenged experimentally with 10^7^ TCID50 of a MERS-CoV strain showed nasal discharges and viral RNA detection that peaked for a week then declined in the second week post-challenge; however, the virus was isolated only in the first week post the experimental challenge, in two different studies [23, 29]. Here, our model showed high viral RNA in the first week that decreased in the second week post natural challenge. Although we did not quantify the infectious virus titre in our model, it showed a similar outcome to the experimental model in terms of viral RNA detection. This model would closely mimic the natural context especially with the lack of biosafety containment labs for large animals in endemic countries as well as that the infectious dose for MERS-CoV is not yet defined for the experimental challenge. Nasal discharges were not observed in naturally infected camels in markets in our study, but it was only observed during the natural challenge model in a confined space. This supports previous studies that nasal discharge in dromedaries, infected with MERS-CoV, manifests in experimental settings [23, 29].

Therefore, we propose that this natural challenge model could be used to assess a MERS vaccine efficacy; to perform this, camels receiving a vaccine as well as camels receiving a placebo (control) should be co-housed in one pen in addition to infectious camels that have Ct values below 25. The infectious camels could be half the number of experimental camels, although further work is required to determine the minimal ratio (or number) of infectious camels for such a model. It should also be noted that there might be some difficulties in conducting this model. First, the number of available infectious camels at the challenge time might not be enough; so the timing of such studies must be carefully planned. Second, infectious camels could vary in terms of viral shedding at the time of challenge; so a single infectious camel might be used as a source of infection to have a more defined model, but this has to be carefully assessed before conducting such studies. Following the natural challenge, nasal swab samples collected daily for 14 days post co-housing (natural challenge) should be used to evaluate RNA levels as well as virus titres in vaccinated camels, control camels, and infectious camels. The difference in virus titres post natural challenge between vaccinated and control camels should be then used to calculate the efficacy rate. Virus titre in infectious camels would also be important to determine how long these animals remain infectious. MERS-CoV strains (isolates) in infectious camels could be different, giving the advantage of assessing vaccines against different circulating strains (or isolates). The exact genetic information of these strains can be analysed and reported along with the efficacy data; although we did not study the genetic sequence of the isolates in the current study. There are several vaccine candidates in development for camels as a one health intervention where vaccinating camels is proposed to reduce spillover infections and transmission into humans. Therefore, in the absence of informative research on what is the MERS viral infectious dose in camels, utilising a natural model of challenge would mimic the natural setting and could accelerate vaccine development.

## Conclusion

This study reports three main conclusions. First, it reports the population and distribution of dromedary camels in the 13 provinces of Saudi Arabia. Second, it reports that the MERS-CoV seroprevalence rate in young dromedaries in Qassim and Jouf provinces of Saudi Arabia in a given time were 94.5 and 77%, respectively. Third, it details the feasibility of utilising MERS-CoV infected camels as a mean to evaluate vaccine efficacy studies in dromedaries; therefore, it establishes a controlled natural challenge model.

## Supplementary information

**Additional file 1: Table S1.** Anti-S1 MERS-CoV antibodies in young camels among different herds in Saudi Arabia. Camels were considered positive if ELISA ratio was 1.1 (confirmed positive) or > 0.8 (borderline/ indeterminate positive). Herds were from Qassim (Q) or Jouf (J) provinces of Saudi Arabia. **Table S2.** RT-qPCR detection for UpE and ORF1a genes of MERS-CoV in experimental and naturally infected camels. Camels were tested in RT-qPCR assay that detects two amplicons: UpE and ORF1a regions. Ct values are reported as detected. Samples that showed no detection were given the arbitrary value of Ct = 40. C indicates a camel followed by a camel number. **Figure S1.** Layout of the research farm. Distances in the layout are shown in metre (m). Each barn has donning and doffing dedicated areas. The study took place in Barn 1.

## Data Availability

All data of this study was analysed and available in this article and its supplementary file.

## Abbreviations

MERS: Middle East Respiratory Syndrome
MERS-CoV: Middle East Respiratory Syndrome coronavirus
KSA: Kingdom of Saudi Arabia
MEWA: Ministry of Environment, Water and Agriculture, Riyadh, Saudi Arabia
KAIMRC: King Abdullah International Medical Research Center, Riyadh, Saudi Arabia
