# Supplementary material for: Challenge infection model for MERS-CoV based on naturally infected camels
Source: Virol J. 2020 Jun 17;17:77. doi: 10.1186/s12985-020-01347-5 (PMC7298446; doi:10.1186/s12985-020-01347-5)
Supplement: Supplementary file 1 — Additional file 1: Table S1. Anti-S1 MERS-CoV antibodies in young camels among different herds in Saudi Arabia. Camels were considered positive if ELISA ratio was 1.1 (confirmed positive) or > 0.8 (borderline/ indeterminate positive). Herds were from Qassim (Q) or Jouf (J) provinces of Saudi Arabia. Table S2. RT-qPCR detection for UpE and ORF1a genes of MERS-CoV in experimental and naturally infected camels. Camels were tested in RT-qPCR assay that detects two amplicons: UpE and ORF1a regions. Ct values are reported as detected. Samples that showed no detection were given the arbitrary value of Ct = 40. C indicates a camel followed by a camel number. Figure S1. Layout of the research farm. Distances in the layout are shown in metre (m). Each barn has donning and doffing dedicated areas. The study took place in Barn 1. [file 12985_2020_1347_MOESM1_ESM.docx]

**Supplementary file**

| Herd | Q1 | Q2 | Q3 | Q4 | Q5 | Q6 | Q7 | J1 | J2 | J3 | J4 | J5 | J6 | J7 | J8 | J9 | J10 | J11 | J12 | Total |
| --- | --- | --- | --- | --- | --- | --- | --- | --- | --- | --- | --- | --- | --- | --- | --- | --- | --- | --- | --- | --- |
| # of screened camels | 9 | 22 | 15 | 21 | 75 | 30 | 61 | 19 | 15 | 7 | 10 | 7 | 7 | 3 | 21 | 6 | 19 | 9 | 6 | 362 |
| # of positive camels | 8 | 16 | 14 | 19 | 74 | 25 | 59 | 18 | 13 | 4 | 5 | 7 | 5 | 1 | 19 | 3 | 17 | 4 | 3 | 326 |
| % of positive camels | 88.89 | 77.27 | 93.33 | 95.24 | 98.67 | 90.00 | 100.00 | 100.00 | 86.67 | 71.43 | 60.00 | 100.00 | 71.43 | 33.33 | 90.48 | 50.00 | 100.00 | 55.56 | 50.00 | 90.06 |
| Individual camels | 6.82 | 5.38 | 5.16 | 5.39 | 5.44 | 6.75 | 1.203 | 4.38 | 1.72 | 0.3 | 0.49 | 4.75 | 5.41 | 1.51 | 3.63 | 5.47 | 4.45 | 0.33 | 1.91 |  |
|  | 5.67 | 5.39 | 4.01 | 2.22 | 5.86 | 6.23 | 1.323 | 3.87 | 1.86 | 0.29 | 3.35 | 2.92 | 5.39 | 0.22 | 0.56 | 0.58 | 4.29 | 2.79 | 3.11 |  |
|  | 1.35 | 2.68 | 2.1 | 6.47 | 6.63 | 3.03 | 1.496 | 3.15 | 2.7 | 3.86 | 4.52 | 2.81 | 4.96 | 0.25 | 1.42 | 1.6 | 2.46 | 1.1 | 0.36 |  |
|  | 2.85 | 5.55 | 5.69 | 5.64 | 6.82 | 6.05 | 1.347 | 1.43 | 5.14 | 2.24 | 0.57 | 3.13 | 1.79 |  | 2.15 | 0.27 | 1.75 | 1.36 | 0.21 |  |
|  | 2.6 | 5.44 | 4.87 | 1.05 | 6 | 4.47 | 1.351 | 1.32 | 3 | 1.4 | 1.55 | 1.65 | 0.79 |  | 1.41 | 0.65 | 2.84 | 0.35 | 0.31 |  |
|  | 1.37 | 6.71 | 6.69 | 1.64 | 4.59 | 5.72 | 1.238 | 1.23 | 4.85 | 1.09 | 0.95 | 1.56 | 1.57 |  | 1.32 | 2.55 | 4.73 | 0.39 | 2.11 |  |
|  | 3.99 | 6.35 | 2.69 | 6.01 | 3.26 | 5.09 | 1.32 | 4.6 | 3.04 | 4.57 | 0.6 | 3.04 | 0.26 |  | 1.46 |  | 1.94 | 2.95 |  |  |
|  | 6.45 | 5.52 | 1.49 | 5.18 | 6.26 | 5.93 | 1.541 | 4.36 | 1.57 |  | 2.64 |  |  |  | 2.32 |  | 1.61 | 1.46 |  |  |
|  | 0.65 | 0.54 | 3.4 | 6.75 | 7.24 | 2.81 | 1.388 | 3.7 | 2.9 |  | 2.49 |  |  |  | 1.22 |  | 3.62 | 0.73 |  |  |
|  |  | 5.56 | 6.1 | 5.71 | 4.63 | 7.3 | 1.257 | 3.66 | 3.9 |  | 0.29 |  |  |  | 2.86 |  | 4.66 |  |  |  |
|  |  | 6.41 | 2.3 | 3.23 | 4.3 | 6.32 | 1.241 | 4.03 | 3.25 |  |  |  |  |  | 4.65 |  | 1.16 |  |  |  |
|  |  | 5.21 | 2.13 | 5.7 | 2.39 | 2.8 | 1.584 | 3.86 | 4.3 |  |  |  |  |  | 4.69 |  | 1.16 |  |  |  |
|  |  | 6.39 | 4.73 | 1.63 | 6.6 | 1.03 | 1.373 | 5.04 | 3.39 |  |  |  |  |  | 2.87 |  | 3.65 |  |  |  |
|  |  | 5.78 | 3.8 | 6.14 | 5.38 | 6.39 | 1.511 | 4.99 | 0.68 |  |  |  |  |  | 0.6 |  | 1.79 |  |  |  |
|  |  | 5.24 | 0.1 | 3.28 | 4.64 | 2.48 | 1.465 | 3.85 | 0.29 |  |  |  |  |  | 5.61 |  | 4.86 |  |  |  |
|  |  | 0.18 |  | 5.79 | 5.8 | 6 | 1.312 | 6.36 |  |  |  |  |  |  | 4.88 |  | 2.38 |  |  |  |
|  |  | 2.62 |  | 5.41 | 5.55 | 6.43 | 1.4 | 1.15 |  |  |  |  |  |  | 5.76 |  | 4.55 |  |  |  |
|  |  | 0.94 |  | 0.76 | 5.38 | 6.56 | 1.426 | 6.02 |  |  |  |  |  |  | 5.65 |  | 4.13 |  |  |  |
|  |  | 0.55 |  | 2.86 | 6.92 | 5.71 | 4.99 | 5.01 |  |  |  |  |  |  | 3.55 |  | 4.73 |  |  |  |
|  |  | 0.49 |  | 4.36 | 5.93 | 0.55 | 5.49 |  |  |  |  |  |  |  | 5.13 |  |  |  |  |  |
|  |  | 1.95 |  | 3.7 | 2.88 | 4.44 | 6.21 |  |  |  |  |  |  |  | 4.87 |  |  |  |  |  |
|  |  | 0.66 |  |  | 6.45 | 1.05 | 5.59 |  |  |  |  |  |  |  |  |  |  |  |  |  |
|  |  |  |  |  | 4.49 | 0.11 | 5.61 |  |  |  |  |  |  |  |  |  |  |  |  |  |
|  |  |  |  |  | 5.59 | 1.33 | 5.14 |  |  |  |  |  |  |  |  |  |  |  |  |  |
|  |  |  |  |  | 5.33 | 1.57 | 5.48 |  |  |  |  |  |  |  |  |  |  |  |  |  |
|  |  |  |  |  | 5.32 | 3.55 | 6.39 |  |  |  |  |  |  |  |  |  |  |  |  |  |
|  |  |  |  |  | 3.31 | 2.41 | 5.76 |  |  |  |  |  |  |  |  |  |  |  |  |  |
|  |  |  |  |  | 5.97 | 1.67 | 5.22 |  |  |  |  |  |  |  |  |  |  |  |  |  |
|  |  |  |  |  | 6.53 | 0.55 | 5.15 |  |  |  |  |  |  |  |  |  |  |  |  |  |
|  |  |  |  |  | 5.37 | 2.44 | 6.57 |  |  |  |  |  |  |  |  |  |  |  |  |  |
|  |  |  |  |  | 6.77 |  | 5.7 |  |  |  |  |  |  |  |  |  |  |  |  |  |
|  |  |  |  |  | 7.11 |  | 6.27 |  |  |  |  |  |  |  |  |  |  |  |  |  |
|  |  |  |  |  | 7.37 |  | 6.08 |  |  |  |  |  |  |  |  |  |  |  |  |  |
|  |  |  |  |  | 6.03 |  | 5.44 |  |  |  |  |  |  |  |  |  |  |  |  |  |
|  |  |  |  |  | 5.8 |  | 5.81 |  |  |  |  |  |  |  |  |  |  |  |  |  |
|  |  |  |  |  | 6.45 |  | 5.92 |  |  |  |  |  |  |  |  |  |  |  |  |  |
|  |  |  |  |  | 4.82 |  | 6.09 |  |  |  |  |  |  |  |  |  |  |  |  |  |
|  |  |  |  |  | 2.73 |  | 2.55 |  |  |  |  |  |  |  |  |  |  |  |  |  |
|  |  |  |  |  | 5.24 |  | 1.75 |  |  |  |  |  |  |  |  |  |  |  |  |  |
|  |  |  |  |  | 6.35 |  | 1.13 |  |  |  |  |  |  |  |  |  |  |  |  |  |
|  |  |  |  |  | 6.51 |  | 5.99 |  |  |  |  |  |  |  |  |  |  |  |  |  |
|  |  |  |  |  | 7.63 |  | 3.78 |  |  |  |  |  |  |  |  |  |  |  |  |  |
|  |  |  |  |  | 7.06 |  | 2.78 |  |  |  |  |  |  |  |  |  |  |  |  |  |
|  |  |  |  |  | 5.75 |  | 1.86 |  |  |  |  |  |  |  |  |  |  |  |  |  |
|  |  |  |  |  | 6.11 |  | 2.81 |  |  |  |  |  |  |  |  |  |  |  |  |  |
|  |  |  |  |  | 5.69 |  | 3.44 |  |  |  |  |  |  |  |  |  |  |  |  |  |
|  |  |  |  |  | 5.74 |  | 3.39 |  |  |  |  |  |  |  |  |  |  |  |  |  |
|  |  |  |  |  | 6.32 |  | 1.77 |  |  |  |  |  |  |  |  |  |  |  |  |  |
|  |  |  |  |  | 6.69 |  | 3.19 |  |  |  |  |  |  |  |  |  |  |  |  |  |
|  |  |  |  |  | 4.39 |  | 3.46 |  |  |  |  |  |  |  |  |  |  |  |  |  |
|  |  |  |  |  | 6.68 |  | 5.8 |  |  |  |  |  |  |  |  |  |  |  |  |  |
|  |  |  |  |  | 6.61 |  | 1.95 |  |  |  |  |  |  |  |  |  |  |  |  |  |
|  |  |  |  |  | 7.42 |  | 2.61 |  |  |  |  |  |  |  |  |  |  |  |  |  |
|  |  |  |  |  | 6.76 |  | 4.78 |  |  |  |  |  |  |  |  |  |  |  |  |  |
|  |  |  |  |  | 4.59 |  | 3.03 |  |  |  |  |  |  |  |  |  |  |  |  |  |
|  |  |  |  |  | 6.27 |  | 6.13 |  |  |  |  |  |  |  |  |  |  |  |  |  |
|  |  |  |  |  | 5.51 |  | 5.43 |  |  |  |  |  |  |  |  |  |  |  |  |  |
|  |  |  |  |  | 5.42 |  | 3.48 |  |  |  |  |  |  |  |  |  |  |  |  |  |
|  |  |  |  |  | 7.95 |  | 1.95 |  |  |  |  |  |  |  |  |  |  |  |  |  |
|  |  |  |  |  | 4.11 |  | 1.41 |  |  |  |  |  |  |  |  |  |  |  |  |  |
|  |  |  |  |  | 5.85 |  | 2.62 |  |  |  |  |  |  |  |  |  |  |  |  |  |
|  |  |  |  |  | 2.65 |  |  |  |  |  |  |  |  |  |  |  |  |  |  |  |
|  |  |  |  |  | 5.54 |  |  |  |  |  |  |  |  |  |  |  |  |  |  |  |
|  |  |  |  |  | 6.29 |  |  |  |  |  |  |  |  |  |  |  |  |  |  |  |
|  |  |  |  |  | 5.43 |  |  |  |  |  |  |  |  |  |  |  |  |  |  |  |
|  |  |  |  |  | 1.82 |  |  |  |  |  |  |  |  |  |  |  |  |  |  |  |
|  |  |  |  |  | 6.77 |  |  |  |  |  |  |  |  |  |  |  |  |  |  |  |
|  |  |  |  |  | 4.8 |  |  |  |  |  |  |  |  |  |  |  |  |  |  |  |
|  |  |  |  |  | 6.46 |  |  |  |  |  |  |  |  |  |  |  |  |  |  |  |
|  |  |  |  |  | 5.99 |  |  |  |  |  |  |  |  |  |  |  |  |  |  |  |
|  |  |  |  |  | 6.07 |  |  |  |  |  |  |  |  |  |  |  |  |  |  |  |
|  |  |  |  |  | 5.3 |  |  |  |  |  |  |  |  |  |  |  |  |  |  |  |
|  |  |  |  |  | 0.78 |  |  |  |  |  |  |  |  |  |  |  |  |  |  |  |
|  |  |  |  |  | 4.67 |  |  |  |  |  |  |  |  |  |  |  |  |  |  |  |
|  |  |  |  |  | 4.76 |  |  |  |  |  |  |  |  |  |  |  |  |  |  |  |

**Table S1: Anti-S1 MERS-CoV antibodies in young camels among different herds in Saudi Arabia.**Camels were considered positive if ELISA ratio was 1.1 (confirmed positive) or >0.8 (borderline/ indeterminate positive). Herds were from Qassim (Q) or Jouf (J) provinces of Saudi Arabia.

| d.p.c. | Experimental camels | | | | | | | | | | Naturally infected camels | | | | | |
| --- | --- | --- | --- | --- | --- | --- | --- | --- | --- | --- | --- | --- | --- | --- | --- | --- |
|  | C_10 | C_10 | C_12 | C_12 | C_16 | C_16 | C_32 | C_32 | C_40 | C_40 | C_101 | C_101 | C_102 | C_102 | C_103 | C_103 |
|  | UpE | ORF1a | UpE | ORF1a | UpE | ORF1a | UpE | ORF1a | UpE | ORF1a | UpE | ORF1a | UpE | ORF1a | UpE | ORF1a |
| 0 | 40 | 40 | 40 | 40 | 40 | 40 | 40 | 40 | 40 | 40 |  |  |  |  |  |  |
| 1 | 40 | 40 | 40 | 40 | 40 | 40 | 24 | 26 | 40 | 40 | 23 | 24 | 25 | 26 | 22 | 24 |
| 2 | 24 | 26 | 34 | 35 | 35 | 37 | 22 | 23 | 28 | 29 | 24 | 25 | 26 | 28 | 23 | 25 |
| 3 | 19 | 21 | 36 | 40 | 34 | 35 | 28 | 29 | 28 | 29 | 29 | 30 | 29 | 31 | 29 | 31 |
| 4 | 18 | 20 | 33 | 35 | 35 | 40 | 29 | 30 | 34 | 35 | 28 | 28 | 30 | 32 | 29 | 30 |
| 5 | 21 | 23 | 35 | 35 | 30 | 32 | 30 | 31 | 33 | 35 | 29 | 30 | 29 | 31 | 31 | 32 |
| 6 | 23 | 24 | 34 | 35 | 34 | 35 | 36 | 36 | 23 | 24 | 29 | 29 | 31 | 33 | 32 | 34 |
| 7 | 28 | 29 | 31 | 33 | 35 | 36 | 35 | 36 | 24 | 26 | 33 | 34 | 40 | 40 | 33 | 34 |
| 8 | 25 | 27 | 26 | 27 | 34 | 35 | 36 | 37 | 24 | 25 | 34 | 35 | 40 | 40 | 34 | 35 |
| 9 | 40 | 40 | 26 | 28 | 23 | 24 | 34 | 35 | 24 | 25 | 32 | 34 | 40 | 40 | 33 | 35 |
| 10 | 40 | 40 | 27 | 39 | 25 | 27 | 33 | 34 | 26 | 27 | 36 | 36 | 40 | 40 | 34 | 35 |
| 11 | 40 | 40 | 29 | 30 | 28 | 27 | 38 | 37 | 27 | 28 | 31 | 31 | 40 | 40 | 34 | 36 |
| 12 | 40 | 40 | 37 | 38 | 33 | 35 | 36 | 40 | 36 | 37 | 34 | 36 | 40 | 40 | 36 | 40 |
| 13 | 40 | 40 | 37 | 40 | 32 | 35 | 40 | 40 | 31 | 33 | 37 | 40 | 40 | 40 | 37 | 38 |
| 14 | 40 | 40 | 35 | 36 | 36 | 38 | 40 | 40 | 33 | 35 | 40 | 40 | 40 | 40 | 40 | 40 |
| 42 | 40 | 40 | 40 | 40 | 40 | 40 | 40 | 40 | 40 | 40 | 40 | 40 | 40 | 40 | 40 | 40 |

**Table S2: RT-qPCR detection for UpE and ORF1a genes of MERS-CoV in experimental and naturally infected camels.**

Camels were tested in RT-qPCR assay that detects two amplicons: UpE and ORF1a regions. Ct values are reported as detected. Samples that showed no detection were given the arbitrary value of Ct=40. C indicates a camel followed by a camel number.

**Figure S1: Layout of the research farm.**Distances in the layout are shown in metre (m). Each barn has donning and doffing dedicated areas. The study took place in Barn 1.
